# Potential Effects of Pomegranate Polyphenols in Cancer Prevention and Therapy

**DOI:** 10.1155/2015/938475

**Published:** 2015-06-09

**Authors:** Eleonora Turrini, Lorenzo Ferruzzi, Carmela Fimognari

**Affiliations:** Department for Life Quality Studies, Alma Mater Studiorum, University of Bologna, 47921 Rimini, Italy

## Abstract

Cancer is the second leading cause of death and is becoming the leading one in old age. Vegetable and fruit consumption is inversely associated with cancer incidence and mortality. Currently, interest in a number of fruits high in polyphenols has been raised due to their reported chemopreventive and/or chemotherapeutic potential. Pomegranate has been shown to exert anticancer activity, which is generally attributed to its high content of polyphenols. This review provides a comprehensive analysis of known targets and mechanisms along with a critical evaluation of pomegranate polyphenols as future anticancer agents. Pomegranate evokes antiproliferative, anti-invasive, and antimetastatic effects, induces apoptosis through the modulation of Bcl-2 proteins, upregulates p21 and p27, and downregulates cyclin-cdk network. Furthermore, pomegranate blocks the activation of inflammatory pathways including, but not limited to, the NF-*κ*B pathway. The strongest evidence for its anticancer activity comes from studies on prostate cancer. Accordingly, some exploratory clinical studies investigating pomegranate found a trend of efficacy in increasing prostate-specific antigen doubling time in patients with prostate cancer. However, the genotoxicity reported for pomegranate raised certain concerns over its safety and an accurate assessment of the risk/benefit should be performed before suggesting the use of pomegranate or its polyphenols for cancer-related therapeutic purposes.

## 1. Introduction

Cancer is the second leading cause of death and is becoming the leading one in old age. It has been estimated that by 2030 the number of new cancer cases will increase by 70% worldwide due to demographic changes alone [1].

The process of cancer development is a consequence of genetic and epigenetic alterations that lead to disruption of basic biological functions, such as cell division, differentiation, angiogenesis, and migration, and transform normal epithelium to preneoplastic lesions and then to invasive carcinoma. The presence of precursor lesions that represent intermediate stages between normal and malignant cells, the slow growth, likely for decades, before symptoms arise and diagnosis is established, a long latency period, and the age-dependent incidence of most cancers indicate that the carcinogenic process occurs during a protracted interval, thus providing the opportunity to block or delay the process, thereby preventing the development of invasive cancer.

Vegetable and fruit consumption is inversely associated with cancer incidence and mortality [2]. Currently, interest in a number of fruits high in polyphenolic compounds has been raised due to their reported chemopreventive and/or chemotherapeutic potential. Pomegranate (*Punica granatum* L.) has been shown to exert anticancer activity, which is generally attributed to its high content of polyphenols including ellagitannins, ellagic acid, and other flavonoids (quercetin, kaempferol, and luteolin glycosides) [3].

The aim of the present review is to critically discuss the cumulative evidence suggesting that pomegranate consumption possesses multiple biological actions and may be helpful in the prevention and therapy of cancer and to provide insight into its anticancer mechanisms.

## 2. Pomegranate Polyphenols

Pomegranate is a fruit-bearing deciduous shrub and the predominant member of two species comprising the Punicaceae family [4]. Fruits are widely consumed fresh and as juice, jam, and wine [5]. Among seed, peel, and juice, the peel is the constituent which possesses higher content of polyphenols [6]. This part of the fruit contains ellagitannins. Punicalagin, a large polyphenol with a molecular weight greater than 1000, is unique to pomegranate and is part of a family of ellagitannins that includes the minor tannins punicalin and gallagic acid. Punicalagin represents the bioactive constituent responsible for >50% of the antioxidant activity of pomegranate juice [7]. Pomegranate also contains other polyphenols, such as anthocyanins (3-glucosides and 3,5-glucosides of delphinidin, cyanidin, and pelargonidin) and flavonols [8]. During the juice processing, the whole fruit is pressed and ellagitannins are released into pomegranate juice in significant levels (over 2 g/L juice) [9].

Ellagitannins are hydrolyzed to ellagic acid in the gut, thus resulting in a prolonged release of this acid into the blood [9]. In humans and different animal models, it has been found that ellagic acid is metabolized by the colon microflora to form urolithins A and B. Urolithins can be absorbed into the enterohepatic circulation, which implies that urolithins are in the systemic bloodstream for a short time and then can be excreted in the urine over 12–56 h, as reported after a single administration of 250 mL of pomegranate juice. The metabolite profile is different among subjects, probably due to differences in colonic microflora, where the ellagitannins are metabolized [10–12]. Ellagic acid and urolithins can circulate in the blood and reach and accumulate in many target organs, including intestine and prostate, where the effects of pomegranate ellagitannins are observed [13, 14].

A study analyzed the bioavailability of ellagic acid hydrolyzed from ellagitannins in 18 volunteers following ingestion of pomegranate juice [15]. The plasma appearance and disappearance rates of ellagic acid have been measured. It was found that punicalagin-derived ellagic acid is transformed into dimethylellagic acid glucuronide in plasma and urine on the day of administration of pomegranate juice. Ellagic acid-derived urolithins appeared in urine after the disappearance of dimethylellagic acid glucuronide about 12 h after the administration of pomegranate juice and persisted for 48 h after pomegranate ingestion.

In another study, 11 volunteers consumed 800 mg capsuled pomegranate extract daily containing 330.4 mg punicalagin and 21.6 mg ellagic acid. Cmax and Tmax for plasma ellagic acid were 33.8 ± 12.7 ng/mL at 1 h after ingestion [16].

## 3. Anticancer Activity of Pomegranate Polyphenols

**Figure 1:**
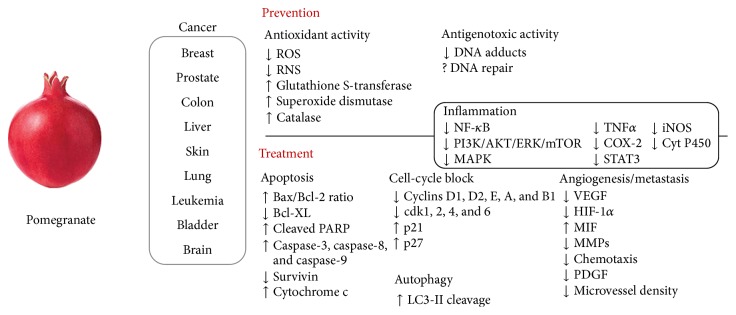
Molecular targets of pomegranate. The arrows reflect changes in protein levels/activities as well as gene expression.

Accumulating evidence suggests that pomegranate targets a broad spectrum of genes and proteins to suppress cancer growth and progression. The anticancer activity of pomegranate can be seen in a chemopreventive and/or chemotherapeutic approach. Extensive mechanistic studies were performed to evaluate the anticancer activity of pomegranate and its therapeutic potential in various preclinical models. Two primary mechanisms that have been reported are cell-cycle arrest and induction of apoptosis. Some authors have also found significant inhibition of other important mechanisms involved in cancer development such as angiogenesis and metastasis.  Figure 1 illustrates the targets and major mechanisms of pomegranate that have been demonstrated in different cancer models.

### 3.1. Cancer Chemoprevention by Pomegranate Polyphenols

Most chemopreventive agents are antioxidant in nature. The antioxidant activity of commercial pomegranate juices obtained from whole pomegranate was evaluated and compared to that of red wine and a green tea infusion. It showed an antioxidant activity three times higher than those of red wine and green tea (18–20 Trolox equivalent antioxidant capacity versus 6–8 Trolox equivalent antioxidant capacity, resp.). The study also compared the antioxidant activity of commercials juices to that of experimental juices obtained from the arils only. The activity was higher in commercial juices than in experimental juices (18–20 Trolox equivalent antioxidant capacity versus 12–14 Trolox equivalent antioxidant capacity, resp.). The higher antioxidant activity of commercial juices can be imputable to their high content of punicalagin (1500–1900 mg/L). Only traces of this compound were detected in the experimental juices [9].

Punicalagin (10–40 *μ*M) has been shown to significantly inhibit oxidative DNA products by about 70% [17]. A pulse radiolysis technique confirmed the antioxidant activity of punicalagin and evidenced that its ability to scavenge reactive oxygen species (ROS) and reactive nitrogen species (RNS) as well as to bind to metal ions such as iron and copper are both involved in its antioxidant activity [18].

Using an electron spin resonance with spin trapping, antioxidant activities have also been reported for the three major anthocyanidins (delphinidin, cyanidin, and pelargonidin) of freeze-dried preparations of a 70% acetone extract of pomegranate [19]. Anthocyanidins exhibited scavenging activity against the OH radical generated by a Fenton reaction through the chelation of ferrous ion. The ID_50_ values (the concentration necessary to scavenge O_2_ by 50%) of delphinidin, cyanidin, and pelargonidin were 2.4, 22, and 456 *μ*M, respectively. In contrast, no effect was observed for anthocyanidins on NO scavenge. Anthocyanidins inhibited H_2_O_2_-induced lipid peroxidation in the rat brain homogenates with ID_50_ values of 0.7, 3.5, and 85 *μ*M for delphinidin, cyanidin, and pelargonidin, respectively. These findings suggest that anthocyanidins contribute to the antioxidant activity of pomegranate.

A study compared the antioxidant activity of pomegranate juice, total pomegranate tannin extract, punicalagin, and ellagic acid (all at 10 *μ*g/mL concentrations) using inhibition of lipid peroxidation and Trolox equivalent antioxidant capacity assays. The trend in antioxidant activity was pomegranate juice > total pomegranate tannin extract > punicalagin > ellagic acid. The highest antioxidant activity of pomegranate juice evidences the multifactorial effects and chemical synergy of the action of multiple compounds of pomegranate compared to single purified active ingredients [3].

Pomegranate and its components have been found to inhibit DNA damage, which represents an event involved in the initiation phase of cancer development. Their antigenotoxic effect is only partly dependent on their antioxidant activity. Pomegranate extract (400, 600, and 800 mg/kg b.w.) significantly reduced cyclophosphamide-induced DNA damage in mouse with concomitant increase in antioxidant enzymes including glutathione S-transferase, superoxide dismutase, and catalase [20]. Furthermore, punicalagin and ellagic acid (50–500 *μ*M) showed significant inhibition of benzo[a]pyrene-induced DNA adducts, with essentially complete inhibition (97%) at 40 *μ*M by punicalagin and 77% inhibition at 40 *μ*M by ellagic acid [21]. The inhibition of benzo[a]pyrene-induced DNA adducts can be due to the inhibition of the cytochrome P450 activity and/or enhancement of phase II enzymes and due to the direct conjugation with benzo[a]pyrene metabolites. Punicalagin has been shown to inhibit cytochrome P450 1A1 [22], which is directly involved in benzo[a]pyrene bioactivation. Thus, this evidence rules out its ability to scavenge benzo[a]pyrene metabolites [21]. However, a different mechanism can be involved in the antigenotoxic activity of ellagic acid. Indeed, the catechol group of ellagic acid has been found to covalently interact with benzo[a]pyrene metabolites [23]. Apparently, the catechol moieties are protected in the punicalagin molecule but, upon hydrolysis, punicalagin is converted in its active metabolite ellagic acid [9]. Therefore, it is likely that* in vivo* punicalagin is indirectly involved in scavenging benzo[a]pyrene metabolites through its catechol containing moieties.

Punicalagin and ellagic acid markedly antagonized the effect of different mutagens (i.e., sodium azide, methyl methanesulfonate, benzo[a]pyrene, and 2-aminoflourine), with maximum inhibition of mutagenicity up to 90% [21].

Inflammation or proinflammatory conditions can activate cellular signaling and lead to the initiation of cancer by inducing DNA damage and epigenetic changes, making inflammatory signaling pathways a target for cancer prevention. Specifically, chronic inflammation of the colon increases the risk for colon cancer [24]. A study explored the effects of pomegranate juice, total pomegranate tannin extract, and punicalagin on inflammatory cell signaling proteins in a human colon cancer cell line [25]. Cyclooxygenase- (COX-) 1 and cyclooxygenase- (COX-) 2 are implicated in the conversion of free fatty acids to prostanoids. In particular, while COX-1 produces prostanoids that regulate normal tissue homeostasis, COX-2 produces prostanoids inducing inflammation. For this reason, COX-2 overexpression is involved in cancer development. The phosphatidylinositol 3-kinase (PI3K)/protein kinase B (AKT)/nuclear factor kappa B (NF*κ*B) pathway positively affect COX-2 expression. Pomegranate juice (6–50 mg/L), total pomegranate tannin extract (30–200 mg/L), and punicalagin (25–200 mg/L) significantly reduced COX-2 protein expression. Consistent with this observation, the juice inhibited AKT activity and reduced NF*κ*B activation. Pomegranate juice was more potent than total pomegranate tannin and punicalagin, as demonstrated by the recorded COX-2 protein reduction (79% for pomegranate juice, 55% for total pomegranate tannin extract, and 48% for punicalagin), thus indicating that other bioactive polyphenols of pomegranate, such as anthocyanins and flavonols, may contribute to its anti-inflammatory activity.

*In vivo* studies support the anti-inflammatory activity of pomegranate and its potential in colon cancer prevention. Pomegranate juice (2504.74 mg gallic acid equivalents/L) suppressed the number of aberrant crypt foci and dysplastic aberrant crypt foci in rats injected with azoxymethane. The effect has been found to be associated with a significant downregulation of proinflammatory enzymes such as nitric oxide synthase and COX-2 at both mRNA and protein levels and targeting of anti-inflammatory miR-126-regulated pathways [26].

Recently, ellagic acid was reported to prevent* in vivo* intestinal inflammation and related cancer development. In a model of dextran sulfate sodium-induced acute and chronic colitis, 2% ellagic acid-supplemented diet slightly improved acute colitis. In the chronic model, ellagic acid significantly inhibited the intestinal inflammation, downregulated inflammatory mediators such as COX-2 and iNOS, and blocked the signaling pathways p38 MAPK (mitogen-activated protein kinase), NF-*κ*B, and IL6/STAT3 (signal transducer and activator of transcription 3) in colon tissue [27].

Promising results and targeting of specific mechanisms have been reported for the prevention of certain cancers including breast, colon, liver and skin by pomegranate and its polyphenols.

#### 3.1.1. Breast

A study performed on mouse mammary organ culture reported a chemopreventive role for pomegranate fractions in breast cancer. Pomegranate fermented juice polyphenols (10 *μ*g/mL) actually caused a 38% inhibition of the incidence of 7,12-dimethylbenz[a]anthracene-induced precancerous mammary lesions. A phenolic fraction of fermented pomegranate juice (10 *μ*g/mL) demonstrated a significantly greater chemopreventive potential, with a 75–90% suppression of lesion formation [28].

Cancer stem cells are thought to be the origin of both primary and secondary breast tumors and thus represent a critical target in breast cancer prevention. Pomegranate extract (5–200 *μ*g/mL) significantly reduced cell viability and blocked cell-cycle progression of a mouse mammary cancer cell line [29], previously characterized as containing a majority of cells possessing stem-cell like characteristics [30], with an IC_50_ (concentration able to decrease cell viability by 50% versus control cultures) of 50 *μ*g/mL [29].

#### 3.1.2. Colon

The Wnt pathway involves a large number of secreted glycoproteins that play a pivotal role in regulating cell fate, differentiation, cell cycle, proliferation, and apoptosis [31]. Aberrant activation of the Wnt signaling has been found to be involved in colon cancer processes [32]. Thus, compounds able to inhibit Wnt signaling may have a role in cancer prevention and treatment. In a 1,2-dimethylhydrazine dihydrochloride-induced rat colon carcinogenesis model, a diet supplemented with 3% (w/w) standardized pomegranate extract inhibited the 1,2-dimethylhydrazine dihydrochloride-induced overexpression of many Wnt-target genes and inhibited tumor incidence. As an example, tumor incidence (66.77%) observed in the 1,2-dimethylhydrazine dihydrochloride group was totally inhibited in the 1,2-dimethylhydrazine dihydrochloride group fed with pomegranate [33]. Also, both ellagic acid and urolithin A inhibited Wnt signaling with IC_50_ (the concentration necessary to inhibit Wnt signaling by 50%) of 19 *μ*g/mL (63 *μ*M) and 9 *μ*g/mL (39 *μ*M), respectively. However, the ellagic acid levels necessary to inhibit Wnt signaling may be near impossible to achieve through regular dietary intake of pomegranate [12, 34]. On the contrary, the inhibition of Wnt signaling achieved by urolithin A is physiologically relevant [35].

Interestingly, significant levels of ellagic acid derivatives and urolithins have been found in human colon tissues from colorectal cancer patients after consumption of pomegranate (900 mg/day for 15 days) [36], thus indicating the induction by pomegranate of a potential molecular preventive environment against colorectal cancer.

The chemopreventive activity of pomegranate on colon carcinogenesis has been found to be also related to its antioxidant activity. Pomegranate peel extract (47 mg (in terms of gallic acid equivalents)/kg of body weight) reduced the incidence of azoxymethane-induced genotoxicity and azoxymethane-induced premalignant lesions by blocking azoxymethane-induced impairment of biochemical indicators of oxidative stress in colonic tissue homogenates [37, 38].

#### 3.1.3. Liver

Pomegranate emulsion has been found to exert chemoprevention of hepatic cancer through antioxidant, antiproliferative, and proapoptotic mechanisms. In particular, Bishayee et al. have found that the emulsion (1 or 10 g/kg) reduces the number and area of *γ*-glutamyl transpeptidase-positive hepatic foci induced in rat by diethylnitrosamine treatment. The effect was associated with the upregulation of several housekeeping genes under the control of Nrf2, such as glutathione S-transferase, NAD(P)H:quinone oxidoreductase 1, and uridine diphosphate-glucuronosyltransferase [39]. Nrf2 acts as a key mediator of NF-*κ*B-regulated inflammatory pathway. Consistent with this observation, the pomegranate emulsion suppressed several inflammatory markers including NO synthase, 3-nitrotyrosine, heat shock protein 70 and 90, COX-2, and NF-*κ*B induced in rats following exposure to diethylnitrosamine [40]. Since pomegranate juice consumption (377 mL/kg b.w.) has been reported to decrease total hepatic cytochrome P450 content as well as cytochrome P4501A2 expression in rodents [41], Bishayee et al. postulated that the chemopreventive effect of pomegranate emulsion may be also ascribable to the attenuation of diethylnitrosamine activation through pomegranate-induced cytochrome P450 inhibition [39]. However, pomegranate juice as a complex food would never reach the liver. Indeed,* in vivo* studies have not demonstrated unequivocally that the consumption of pomegranate juice may interfere with drug metabolism and clearance [42].

Pomegranate emulsion (1 or 10 g/kg) also reversed hepatic proliferation induced in rat by diethylnitrosamine treatment and induced apoptosis through the upregulation of the proapoptotic protein Bax and the downregulation of the antiapoptotic protein Bcl-2. Canonical NF-*κ*B and Wnt/*β*-catenin pathways, two interconnected molecular circuits implicated in liver physiology and pathology through regulation of proliferation, differentiation, survival, inflammation, and regeneration [43], have been shown to be involved in the above reported effects and thus in the hepatocarcinogenesis prevention by pomegranate [44].

#### 3.1.4. Skin

Excessive exposure to solar UVB and, to a lesser extent, UVA radiations is the major cause of a variety of cutaneous disorders including skin cancers. In normal human epidermal keratinocytes exposed to UVB, pretreatment with pomegranate fruit extract (10–40 *μ*g/mL) inhibited UVB-dependent activation of NF-*κ*B and UVB-mediated phosphorylation of ERK1/2, JNK1/2 and p38 protein [45], an important group of MAPK pathway signaling proteins that regulate cell proliferation, differentiation, and survival. Furthermore, pomegranate fruit extract (5–60 mg/L) protected human skin fibroblasts from cell death following UV exposure through a reduced activation of the proinflammatory transcription factor NF-*κ*B, downregulation of proapoptotic caspase-3, and an increased G0/G1 phase associated with DNA repair. However, only higher polyphenolic concentrations (500–10,000 mg/L) were able to significantly decrease UV-induced reactive oxygen species levels and increase intracellular antioxidant capacity [46]. Pomegranate exhibited protective effects also against UVA. Pretreatment of normal human epidermal keratinocytes with pomegranate fruit extract (60–100 *μ*g/mL) actually reduced different cellular pathways including phosphorylation of STAT3, AKT, ERK1/2, mTOR, and p70S6K [47]. The ability of pomegranate fruit extract (0.2%, w/v) to enhance the repair of UVB-mediated DNA damage (i.e., cyclobutane pyrimidine dimers and 8-oxo-7,8-dihydro-2′-deoxyguanosine) recorded in hairless mice [48] contributes to its skin photoprotective activity.

Some studies were designed to extend the skin chemopreventive potential of pomegranate to an* in vivo* situation. Oral feeding of pomegranate fruit extract (0.2% w/v) inhibited skin edema, hyperplasia, infiltration of leukocytes, lipid peroxidation, and hydrogen peroxide generation in the murine skin following UVB exposure. Moreover, it protected against UVB-induced DNA damage and increased p53 and cyclin kinase inhibitor p21 protein levels [48].

Further studies support the ability of pomegranate to prevent skin cancer. In particular, topical application of pomegranate fruit extract (2 mg/mouse) resulted in a significant inhibition of several markers of skin tumor promotion including skin edema and hyperplasia, epidermal ornithine decarboxylase activity, and protein expression of ornithine decarboxylase and COX-2 [49].

The cancer skin chemopreventive effect of pomegranate has been found to be potentiated by diallyl sulfide, a component of garlic. Using a two-stage mouse skin tumorigenesis, pomegranate fruit extract and diallyl sulfide synergistically delayed tumor onset and incidence associated with apoptosis induction and decreased expression of proteins involved in MAPK pathway [50].

### 3.2. Cancer Chemotherapy by Pomegranate Polyphenols

#### 3.2.1. Breast Cancer

The inhibition of estrogen activity represents a consolidated strategy for the treatment of hormone dependent breast cancer. This strategy includes the antagonism to the estrogen receptor (ER) or the inhibition of estrogen synthesis. The biosynthesis of estrogen is mediated by aromatase enzyme, which converts testosterone to estradiol, a hormonal biomarker that directly correlates with the occurrence of breast cancer.

Pomegranate ellagitannin-derived compounds exhibit antiaromatase activity and affect the growth of breast cancer cells* in vitro* [51]. The antiaromatase activity was tested for ellagic acid, gallic acid, urolithins A and B, and their methylated, acetylated, and sulfate derivatives (all tested at the concentration of 47 *μ*mol/L) and then their antiproliferative potential was tested in human breast cancer cells overexpressing aromatase (MCF-7aro) at concentrations of 2.35 and 4.7 *μ*mol/L. Urolithin B showed the highest antiaromatase activity (about 60% of inhibition) and, as expected, had the highest antiproliferative effect on MCF-7aro [50]. The marked efficacy of urolithin B might be due to its better absorption in cells compared to the other components [52]. Of note, when pomegranate polyphenols were tested together, a higher antiaromatase inhibition has been found, thus suggesting a synergistic effect of the combination. For example, Kim and colleagues demonstrated that polyphenols from pomegranate fermented juice and aqueous pericarp extract (0.02 *μ*g/mL) induced a 60–80% aromatase inhibition [53]. Taken into account that 47 *μ*mol/L (i.e., about 10 *μ*g/mL) of urolithin B are necessary to inhibit the aromatase activity of 40%, it is evident that different compounds should act together for inducting the 60–80% aromatase inhibition reported for 0.02 *μ*g/mL of pomegranate fermented juice and aqueous pericarp extract.

The inhibition of proliferation induced by ellagitannin-derived compounds is also due to a direct antagonism to ER, an antiaromatase-independent mechanism. As breast tumors require estrogen to grow, the 55% inhibition of the estrogenic activity of 17-*β*-estradiol by lyophilized fresh pomegranate juice (10 mg/mL) in a yeast estrogen screen is relevant [53]. Many pomegranate components, such as luteolin, kaempferol, quercetin, and naringenin, possess the ability to inhibit the estrogenic action of 17-*β*-estradiol, through competitive binding to ER [54].

Further studies were devoted to test the antiproliferative effect exerted by polyphenols from fermented pomegranate juice and pericarp (starting from 50 *μ*g/mL). A major antiproliferative effect was observed on ER+ cell line (MCF-7), compared to ER− cells (MDA-MB-231) and normal human breast epithelial cells (MCF-10A) [53]. The antiproliferative effect of fermented juice polyphenols was approximately 2-fold higher than that of fresh juice polyphenols [53]. This different activity can be attributed to the fermentation process, which induces breakage of polyphenol-sugar complexes and thus release of free polyphenols [55, 56].

Several studies evidenced the proapoptotic activity of pomegranate. The methanolic extract of pomegranate, whose total phenolic content was 331.28 mg of gallic acid equivalents/g, induced apoptosis on human breast cancer cells at treatment concentration of 100 *μ*g/mL. Additionally, starting from 200 *μ*g/mL, it upregulated the expression of Bax and downregulated Bcl-2 expression [57].

The proapoptotic effect of pomegranate extracts (40 *μ*g/mL) was also investigated on human breast cancer cells in combination with genistein [58], a phytoestrogen isoflavon able to induce apoptosis in ER+ breast cancer cells [59]. Apoptosis induction and cell-growth inhibition of the combination was significantly higher than that of single compounds [58]. These results suggest that the association of genistein and pomegranate could be useful in association with anticancer drugs used for breast tumor.

Tamoxifen is often used against ER+ breast cancer and acts as an ER modulator in breast tissues. Pomegranate fruit extracts (300 *μ*g/mL) additively enhanced tamoxifen-induced inhibition of mitogenic action of estrogen, tamoxifen-induced inhibition of cell cycle, and tamoxifen-induced apoptosis in human breast cancer cells [60]. Furthermore, pomegranate fruit extract restored sensitivity to tamoxifen in tamoxifen-resistant breast tumor cells [60].

Cell-cycle progression is a complex mechanism that leads cells to duplicate their DNA content between phase G1, through phases S and G2, before reaching the cellular division in M phase. The passage among cell-cycle phases is strictly regulated by cyclins and cyclin-dependent kinase (cdk) complexes. A standardized pomegranate extract inhibited cell growth by inducing cell-cycle arrest in G2/M phase followed by the induction of apoptosis on human breast cancer cells. Treatment of cells with 50 *μ*g/mL of extract for 72 and 96 h resulted in 50% and 80% inhibition of cell growth, respectively [61]. As emerged by comparing the antiproliferative effect of pomegranate extract with that of Trolox and N-acetylcysteine at doses containing equivalent antioxidant activity as the extract, cell-growth inhibition cannot be totally attributed to the consolidated antioxidant properties of pomegranate [61]. The proapoptotic and antiproliferative activity of pomegranate extract was confirmed by data obtained through DNA microarray analysis, which suggests that the cellular effects induced by the extract are related to its ability to affect DNA repair pathway. Pomegranate extract actually downregulated many genes involved in homologous recombination. This effect sensitizes cells to DNA double-strand breaks, a genotoxic event playing a critical role in cancer cell survival [61]. Other anticancer drugs, such as bortezomib, imatinib, and histone deacetylase inhibitors, target homologous recombination [62–64]. This evidence might encourage further studies to test the effect of pomegranate extract in association with the anticancer drugs above reported.

In a mouse mammary cancer cell line derived from MMTV-Wnt-1 mammary mouse tumors (WA4), an arrest in G0/G1 phase was observed after treatment with a commercially available and HPLC-standardized pomegranate extract (Pomella) [29]. The extract exerted a cytotoxic effect also in quiescent WA4 cells, with a dose- and time-dependent activation of caspase-3 that suggests apoptotic cell death (IC_50_ = 200 *μ*g/mL). The cytotoxic effect of the extract has been attributed to its components, such as ellagic acid, ursolic acid, and luteolin, for which an IC_50_ between 5 and 10 *μ*M was recorded. Among these phenolic compounds, ursolic acid exerted the most potent inhibitory effect on cell viability and proliferation. The WA4 cell line is characterized by a majority of stem cells [29]. Therefore, the cytotoxic effects of pomegranate extract on WA4 cells are particularly relevant due to the role of stem cells in primary and secondary breast cancer onset.

Angiogenesis represents the physiological process of new vessels formation and has a crucial role in the development and spread of tumors [65]. Tumor microenvironment requires angiogenesis for supplying oxygen and nutrition, shedding waste metabolites, and allowing tumor growth and progression. Therefore, antiangiogenesis represents a useful anticancer strategy. Accordingly, there are much* in vitro* and* in vivo* evidence on the antiangiogenic properties of pomegranate extract.

Toi and colleagues demonstrated that pomegranate fermented juice polyphenols possess antiangiogenic properties mediated by VEGF downregulation in ER+ breast cancer cells (MCF-7) and immortalized normal human breast epithelial cells (MCF-10A) and upregulation of the angiogenic suppressor migration inhibitory factor (MIF) in ER− breast cancer cells (MDA-MB-231). Additionally, on human umbilical vein endothelial cells (HUVECs), pomegranate extracts (10 *μ*g/mL) showed antiproliferative effects and inhibited tube formation. The same inhibitory activity was confirmed in an* in vivo* angiogenesis model of chicken chorioallantoic membrane, where the inhibition of vessel formation by fermented juice was observed [66].

The matrix metalloproteinases (MMPs), an enzymatic family characterized by a zinc ion in the active site, are critical for maintaining tissue allostasis [67]. An alteration in the regulation of MMPs is common in several tumors and leads to the proteolysis of the extracellular matrix (ECM), a well-established prometastatic mechanism [68, 69]. Multiple MMPs have been associated with tumor progression. As an example, MMP-2, -7, -9, and -11 were identified as responsible for tumor progression in MMP-deficient mice [70]. In the antimetastatic and anti-invasive effect attributed to pomegranate, the inhibition of MMPs represents a fundamental mechanism of action. A polyphenolic extract of pomegranate (0–50 mg/L) inhibited MMP-1, -3, and -13 at posttranscriptional and posttranslational level in human chondrocytes even when administered after treatment with IL-1*β* [71], an interleukin able to induce upregulation of MMPs, especially MMP-1 and -13 [72]. Furthermore, pomegranate juice (1-2 *μ*L/0.1 mL/well) showed the ability to preserve human reconstituted skin from the formation of MPPs (MMP-1, -2, -3, -7, and -9) when exposed to UVB radiations [73]. Through a semiquantitative RT-PCR, Sreeja and colleagues reported the ability of the pomegranate pericarp extract (160 *μ*g/mL) to downregulate the transcription of MMP-9 in MCF7 [74].

#### 3.2.2. Prostate Cancer

Prostate cancer represents the most common cancer in man. It is estimated for 2014 that in US 233 000 men will develop prostate cancer and 29480 will die from prostate cancer [75]. Nowadays there is lack in the treatment of this disease except for the surgery and radiation approach applicable for prostate cancer in early stage. Among all natural compounds studied for the prevention and/or treatment of prostate cancer, pomegranate has been proven to possess relevant* in vitro* and* in vivo* beneficial effects.

Tissue androgens play a pivotal role in facilitating signaling pathways mediated by androgen receptor leading to prostate cancer progression. During the initial phase, prostate cancer is an androgen-regulated disease that subsequently evolves in an androgen-independent one [76]. Therefore, androgens and their receptors are essential for prostate cancer development, growth, and progression. Treatment of androgen-dependent LNCaP, androgen-independent LNCaP-AP, an engineered cell line overexpressing androgen receptor, and androgen-independent DU145 with pomegranate extract (50 *μ*g/mL) and pomegranate juice (powder form, 100 *μ*g/mL) resulted in the reduction of the expression levels of genes involved in androgen biosynthesis, such as 5*α*-reductase type I and 3*β*-hydroxysteroid dehydrogenase type II [77]. Furthermore, a recent study investigated the effects of an ethanolic pomegranate extract on androgen biosynthesis pathways on two human prostate cancer cell lines as well as a murine model of prostate cancer (conditional PTEN knockout model, representing a comprehensive model for tumor initiation and progression through all stages of prostate cancer to metastatic disease). Pomegranate extract reduced the concentration of testosterone and dihydrotestosterone generated through steroid biosynthesis pathways and decreased the expression of prostate-specific antigen (PSA).* In vivo* data evidenced that pomegranate administered orally in drinking water at a concentration of 0.17 g/L significantly decreased dehydroepiandrosterone, testosterone, and pregnenolone. The decreased ratios indicating the reduced percentages between samples and controls were 42.1%, 80.3%, and 36.5%, respectively [78].

Several groups reported the ability of pomegranate juice or extract to inhibit prostate cancer cell growth* in vitro*. In particular, fermented juice polyphenols and pericarp polyphenols showed cell death induction in three prostate cancer cell lines (PC3, DU145, and LNCaP) [79]. PC3 is an androgen-independent cell line characterized by a high invasive and metastatic potential; DU145 is also an androgen-independent cell line, highly proliferative but with a moderate metastatic potential; LNCaP is an androgen-dependent cell line characterized by functional androgen receptors and the ability to secrete PSA. Fermented juice and pericarp polyphenols in concentration between 20 and 100 *μ*g/mL have been found to inhibit proliferation and induce apoptosis in all three prostate cancer cell lines [79].

In cell-cycle progression, the transition between G1 and S phase is regulated by cyclin D and E. Furthermore, a critical role is played by cdk-cyclin complexes inhibitor such as p21 and p27 [80]. Malik and colleagues identified in the modulation of cdk the main mechanism involved in the proapoptotic and antiproliferative potential of pomegranate [81]. In particular, the pomegranate extract (10–100 *μ*g/mL), obtained from the squeeze of the peeled edible portion of the fruit in 70% acetone/30% distilled water, inhibited PC3 growth through a block in the G1 phase, which was evoked by the modulation of regulatory molecules involved in cell-cycle progression and, in particular, in the G1-S transition. Pomegranate fruit extract actually downregulated cyclins D1, D2, E, cdk2, cdk4, and cdk6 and upregulated p21 and p27 [81, 82]. Furthermore, the induction of apoptosis by pomegranate in PC3 cells was associated with an increased expression of cleaved PARP and Bax and the inhibition of Bcl-2 [81].

Wang and colleagues reported the ability of pomegranate extract (POM Wonderful, Los Angeles, CA, USA), a standardized pomegranate extract containing 37–40% punicalagin and 3.4% free ellagic acid, to induce a potent* in vitro* cytotoxic effect on metastatic castration-resistant prostate cancer cell lines such as C4-2 (IC_50_ = 42 *μ*g/mL), PC3 (IC_50_ = 78 *μ*g/mL), and ARCaP_M_ (IC_50_ = 161 *μ*g/mL) [83].

The inhibitor-of-apoptosis family member surviving is highly expressed in many cancers and plays a pivotal role in the regulation of cell death, tumor progression, and chemotherapy resistance. In prostate cancer, survivin is frequently overexpressed and associated with poor clinical outcome and resistance to hormone therapy, chemotherapy, and radiation therapy. Based on these pieces of evidence, survivin represents an innovative and promising target for the treatment of prostate cancer [84]. Pomegranate extract (35–150 *μ*g/mL) has been shown to reduce survivin protein and gene expression and modulate its survivin pathway in prostate cancer cells (C4-2, PC3, ARCaP_M_). STAT3 is an inducer of survivin gene expression. Pomegranate extract actually inhibited STAT3 phosphorylation at Ser727, thus leading to the inactivation of STAT3-dependent transcription of survivin. Furthermore, pomegranate extract (i.p. 60 mg/kg; 3 times/week for 12 weeks) induced apoptosis, retarded cell growth, inhibited survivin, and increased the efficacy of docetaxel (5 mg/kg once a week) in prostate cancer cell-transplanted BALB/c nu/nu mice [83].

Inhibition of prostate cancer cell growth by pomegranate was also reported in immunodepressed mice subcutaneously or orthotopically transplanted with human androgen-dependent CWR22Rv1 prostate cancer cells. Oral administration of pomegranate fruit extract (0.1% and 0.2%, w/v) in drinking fluid to athymic nude mice implanted with androgen-dependent prostate cancer cells (CWR22Rv1) resulted in a significant inhibition in tumor growth concomitant with a significant reduction in secretion of PSA in the serum. As an example, 8 days after cell inoculation, solid tumors were observed in animals receiving water as a drinking fluid. This latency period was prolonged to 11–14 days in animals receiving pomegranate fruit extract. The highest inhibitory effects were observed in animals receiving 0.2% pomegranate fruit extract [81]. Likewise, pomegranate extract (0.8 mg/mouse; ca. 10 times the dose administered to a 70 kg man) induced the same inhibitory effect on androgen-dependent LAPC4 cells implanted in severe combined immunodeficient mice (SCID) [14]. When implanted subcutaneously in murine models, LAPC4 cells produce androgen-dependent tumors; after mouse castration, these cells regrow losing their dependence from androgens [76]. To better predict the effect of pomegranate extract in the clinical response to androgen deprivation caused by castration, Rettig and colleagues used a LAPC4 xenograft model. The authors demonstrated that pomegranate extract is able to delay the growth of LAPC4 androgen-independent tumor through the induction of apoptosis and the inhibition of cell proliferation [85]. NF-*κ*B pathway is one of the main inflammatory signaling pathways involved in cancer development. The constitutive activation of NF-*κ*B pathway is commonly observed in primary prostate cancer and constitutes a risk factor for the development of relapse after radical prostatectomy [86, 87]. NF-*κ*B modulates the transcription of several genes involved in the apoptotic and proliferation process. Rettig and colleagues identified in the inhibition of NF-*κ*B a critical event involved in the induction of apoptosis and inhibition of cell proliferation by pomegranate extract in LCAP4 cells [85].

The inhibition of prostate cancer growth was confirmed in a very recent study by using the murine transgenic adenocarcinoma of the mouse prostate (TRAMP) model. The TRAMP model is widely used in classical chemoprevention protocols since closely mirrors the pathogenesis of human prostate cancer. In a very recent study [88], TRAMP mice received 0.1 and 0.2% pomegranate fruit extract, equivalent to 250 and 500 mL of pomegranate juice, in drinking water, starting at 6 weeks and examined at 12, 20, and 34 weeks of age. Pomegranate fruit extract supplementation significantly inhibited the development of advanced prostate cancer and its metastasis and doubling the overall survival time. As an example, in water-fed group, 100% mice developed palpable tumors by 20 weeks compared with only 70 and 50% recorded at 34 weeks in the 0.1% and 0.2% pomegranate fruit extract-supplemented mice, respectively. 0.1 and 0.2% pomegranate fruit extract supplementation increased median life expectancy of 30 and 49 weeks, respectively, compared with median survival of 43 weeks recorded in water-fed mice. Of note, in tumors and prostate tissues, supplementation with pomegranate fruit extract resulted in a significant inhibition of mTOR pathway, a master switch of cellular catabolism and anabolism, and thereby a critical regulator of cell growth and proliferation [89].

IGF-I is upregulated in prostate cancer, where it represents a potent mitogen and prosurvival factor and an epidemiologically risk factor for the development of prostate cancer. IGF-I is regulated by 6 different binding proteins (IGFBP). IGFBP-3 is the most abundant in serum and possesses the ability to inhibit IGF-I and stimulate the induction of apoptosis and the inhibition of cell growth [90]. Pomegranate extract (10 *μ*g/mL) in association with IGFBP-3 (1 *μ*g/mL) synergistically induced apoptosis and additively reduced cell proliferation in LNAP4 through the suppression of AKT/mTOR signaling pathway and the increased phosphorylation of JNK [91].

Proof of the antitumor effect of pomegranate on prostate cancer cells have been reported also for some single components of pomegranate. Ellagic acid and urolithin A induced cell-growth inhibition and apoptosis in DU145 and PC3 cells [92].

Hypoxia exerts a key role in the induction of angiogenesis in cancer mainly through the regulation of HIF-1*α* (hypoxia inducible factor 1*α*). Pomegranate extract (2.5 *μ*g/mL) exhibited antiangiogenic activity in hypoxic conditions. Both in human prostate cancer cell (LNCaP) and in HUVEC, an inhibition of cell proliferation was observed. VEGF and HIF-1*α* protein levels became downregulated in hypoxic conditions and this observation supports a direct effect against angiogenesis of pomegranate extract [93]. In SCID mice injected subcutaneously with human prostate cancer cells (LAPC4), pomegranate extract was orally administered (0.8 mg pomegranate extract dissolved in 0.05 mL PBS for 5 days per week). After 4 weeks of treatment, a decrease in tumor growth, microvessel density, HIF-1*α* and VEGF expression have been found. Pomegranate extract decreased HIF-1*α* expression, which induced VEGF peptide level downregulation, as already shown in the* in vitro* model. A decreased tumor vessel density and a decreased prostate cancer xenograft size compared to vehicle treatment have been observed [93]. Thus, the antiangiogenic effect can contribute to the inhibition of tumor growth induced by pomegranate extract treatment.

Furthermore, based on the well-known role of inflammation in various types of cancer and the codependence between angiogenesis and inflammation [94], the anti-inflammatory effects of ellagitannins, such as the inhibition of NF-*κ*B and COX-2 [95], can be involved in the inhibition of angiogenesis. A proteomic study exploring the effect of pomegranate fruit juice (7.5 mg/mL) on prostate cancer cells (DU145) identified other targets potentially involved in the antiangiogenic activity of pomegranate. Lee and coworkers observed a significant downregulation of prolidase gene expression [96]. Prolidase can induce the expression of HIF-1*α* and VEGF and is, therefore, involved in the angiogenic process. Taken together, these observations suggest that the inhibition of prolidase might contribute to the inhibition of angiogenesis and invasion mediated by pomegranate extracts.

Evidence on the antiangiogenic effect of pomegranate is strengthened by the activity of single compounds present in pomegranate, such as ellagic acid. Indeed, ellagic acid (10 *μ*M) has been shown to inhibit the phosphorylation of VEGF receptor and platelet-derived growth factor (PDGF) receptor in muscle cells with consequent inhibition of the signaling of these receptors, including angiogenesis [97].

Cell invasion and migration represent two key steps for tumor metastasization process. Several studies have found the antimetastatic and anti-invasive potential of pomegranate and its polyphenols. Inhibition of proinflammatory chemokines, chemotaxis, and arachidonic acid and hyaluronan metabolism represent some of the main mechanisms modulated by pomegranate treatment in breast and prostate cancer cells.

A marked inhibition of cell invasion induced by pomegranate was reported by several groups in breast cancer and prostate cancer cell lines, through the evaluation of cell passage across a Matrigel membrane. An anti-invasive effect was evoked in PC3 cells by pomegranate fermented juice polyphenols and pomegranate pericarp polyphenols [79], and an increased effect was reported when these pomegranate derivatives were used in association (equally combined with a total concentration of 3 *μ*g/mL) [98]. Notably, ellagic acid (20 and 50 *μ*M) showed the ability to induce a reduction of PC3 invasion and migration.

Arachidonic acid turnover plays a pivotal role in the process of cancer cell survival and invasiveness [99]. Phospholipase A2 (PLA2) induces the release of arachidonic acid from membrane phospholipids and COX metabolizes arachidonic acid in prostaglandins and thromboxanes. PEG2 represents one of the most important metabolites of arachidonic acid. Several studies attributed to PEG2 the ability to promote cancer cell survival and invasion* via* PI3K/AKT pathway activation [100]. In prostate cancer, arachidonic acid turnover is highly increased (10 times compared to healthy cells), and the concentration of cytosolic PLA2 is increased too [101]. Lansky and colleagues associated the anti-invasive effect expressed by fermented juice and pericarp polyphenols (alone and in association) to their ability to modulate the arachidonic acid pathway. In particular, they reduced the PLA2 mRNA expression in PC3 cells (50% by fermented juice and pericarp polyphenols and 80% by their combination, resp., compared to the control) [98]. Moreover, Lansky et al. reported an inhibition of PC3 invasion induced by isolated pomegranate compounds, alone and in combination (at same gross dosage 4 *μ*g/mL), including ellagic acid, caffeic acid, luteolin, and punicic acid [102].

Another important factor involved in the antimetastatic effect of pomegranate is its ability to modulate the hyaluronan metabolism. Hyaluronan, an anionic nonsulfated glycosaminoglycan overexpressed in many tumors, plays a crucial role in tumor progression, supporting cell migration, invasion, and metastasis [103]. Hyaluronan exerts its tumor promoting activity by binding to cell-surface receptor, in particular the hyaluronan-mediated motility receptor (HMMR). This interaction promotes the transduction of many intracellular signals leading to a series of cellular responses, such as protein kinase C, focal adhesion kinase, MAPK, PI3K, tyrosine kinases, RAS, and NF-*κ*B production [104]. A gene expression analysis performed on PC3 cells treated with pomegranate juice and some of its components including luteolin, ellagic acid, and punicic acid (alone and in combination) revealed their ability to downregulate the expression of HMMR [82, 105]. Since the same pomegranate products are responsible for the inhibition of invasion in the same* in vitro* model, this poses the modulation of HMMR and in general of hyaluronan signaling pathway as a crucial mechanism in the inhibition of cancer progression evoked by pomegranate.

Chemokines are small proinflammatory chemoattractant cytokines and represent the main regulators of cell trafficking and adhesion [106]. In particular, the chemokine CXCL12, known as stromal cell-derived factor-1 (SDF1*α*), binds primarily to the chemokine receptor 4 (CXCR4). The CXCL12/CXCR4 axis is responsible for the regulation of many intracellular signals involved in several pathways such as chemotaxis, cell survival and/or proliferation, increase in intracellular calcium, and gene transcription. The CXCL12/CXCR4 axis plays a critical role in tumor progression, in particular in the angiogenesis, metastasis, and survival processes [107]. Wang and colleagues reported that pomegranate juice and its constituents were able to inhibit chemotaxis acting on CXCL12 in different hormone-dependent and -independent prostate cancer cells (DU145, PC3, and LNCaP) [82, 105]. Furthermore, the inhibition of CXCL12/CXCR4 axis was confirmed by the evaluation of the effect of luteolin, punicic acid, and ellagic acid combination (i.p., 64 *μ*g/component/day) on the formation of metastasis and the expression of CXCR4 on luciferase expressing human prostate cancer cells (PC-3M-luc) implanted in SCID mice. The combination completely inhibited the formation of metastasis and significantly decreased CXCR4 protein levels. Moreover, the combination induced a downregulation of proteins involved in the CXCR4 downstream signaling (G*α*_13_, PI3K, and p-AKT) [108]. A modulation of chemotaxis toward SDF1*α*, a chemokine attracting breast cancer cells to the bone, by pomegranate juice (1%) and the combination of luteolin, punicic acid, and ellagic acid (2 and 4 *μ*g/mL), was reported also in ER+ and ER− breast cancer cells by Rocha and colleagues, associated with inhibition of cell growth and migration and induction of cell adhesion [109].

Other effects induced by pomegranate that strengthen the correlation between this fruit and the inhibition of tumor progression were identified in its ability to inhibit cancer cell migration and enhance adhesion, two crucial cellular processes for cancer metastasis. In particular, pomegranate juice and a combination of luteolin, punicic acid, and ellagic acid reduced cell migration through the downregulation of several genes such as type I collagen, tenascin C, and chimerin 1 in prostatic cancer cells (PC3) [82], HMMR, collagen type I alpha1 (COL1A1), anillin (ANLN), and nexilin (NEXN) in breast cancer cells (MCF7) [109]. On the other hand, pomegranate juice and the combination upregulated genes involved in cell adhesion, in particular* E*-cadherin in PC3 cells, claudin 1 (CLDN1) in MCF7 cells, and intercellular adhesion molecule 1 (ICAM1) and myristoylated alanine-rich protein kinase C (MARCKS) in both tumor models [82, 109].

Furthermore, pomegranate juice induced upregulation of anti-invasive miRNAs including miR-335 (regulating* COL1A1*), miR-205, miR-200, and miR-126 and downregulated proinvasive miRNAs such as miR-21 (regulating* MARCKS*) and miR-373 in DU145 cells [82].

On the whole, the above reports unequivocally suggest the potential role of pomegranate in prostate cancer treatment. In this light, the ability of ellagitannins, representing the most abundant polyphenols present in pomegranate juice, and their bioactive metabolites (i.e., urolithin A) to concentrate in mouse prostate tissue after intraperitoneal administration [14] and in the human prostate gland upon consumption of pomegranate juice or extract [110, 111] would represent* per se* a relevant phenomenon in a therapeutic setting and warrants future human tissue bioavailability studies and clinical studies in men with prostate cancer.

#### 3.2.3. Lung Cancer

Pomegranate fruit extract, obtained from acetone extraction of edible seeds, showed anticancer effects on lung tumor both* in vitro* and* in vivo*. The antiproliferative activity of pomegranate fruit extract (50–150 *μ*g/mL) was tested both in adenocarcinomic human alveolar basal epithelial cells (A549) and in normal human bronchial epithelial cells (NHBE) showing a minimal effect in normal cells and a decrease in cell viability up to 47% at the highest tested pomegranate concentration on A549 cells [112]. Furthermore, pomegranate fruit extract treatment of A549 induced a strong cell-cycle arrest in G1 phase, with a 72% cell in G1 at the highest tested concentration (150 *μ*g/mL). The cell-cycle block induced by pomegranate fruit extract was associated with a marked and dose-dependent induction of protein responsible for the transition from G1 to S phase, such as WAF1/p21 and KIP1/p27. Accordingly, pomegranate fruit extract treatment has been found to downregulate cyclins D1, D2, and E and also cdk2, cdk4, and cdk6, all involved in cell-cycle regulation of G1 phase. Moreover, pomegranate fruit extract downregulated (Ki-67 and PCNA) and inhibited (MAPK, PI3K/AKT, and NF-*κ*B/p65) different proliferation markers [112]. The anticancer effect of pomegranate fruit extract reported on lung cell cultures was confirmed by* in vivo* data. In athymic mice implanted with adenocarcinomic human alveolar basal epithelial cells, pomegranate fruit extract (0.1 and 0.2%, w/v) administered by drinking fluid prolonged the latency period for tumor appearance from 15 to 19 days after cell inoculation [112]. Similar results have been found in two different bioassays of lung tumorigenesis, where benzo[a]pyrene and N-nitroso-tris-chloroethylurea were used to induce lung tumors. Oral consumption of 0.2% pomegranate fruit extract (w/v) in drinking water induced a significant reduction of lung tumor multiplicities. The highest tumor reduction was 61.6% in the benzo[a]pyrene + pomegranate fruit extract group at 140 days and 65.9% in the N-nitroso-tris-chloroethylurea + pomegranate fruit extract group at 240 days. Those effects were associated with the inhibition of several markers of cell proliferation and angiogenesis including phosphorylation of MAPKs, activation of NF-*κ*B, Ki-67, and proliferating cell nuclear antigen, VEGF (vascular endothelial growth factor) [113].

Taken together, these findings indicate pomegranate fruit extract as a promising chemotherapeutic agent in non-small cell lung cancer.

*In vivo* evidence of the antiangiogenic effect of pomegranate fruit extract 0.2% (w/v) was reported in two A/J mice lung tumors models. In the first one, lung tumor was inducted by benzo[a]pyrene and the second one by N-nitroso-tris-chloroethylurea. NO is a genotoxic reactive nitrogen species synthesized* in vivo* by NO synthases. The inducible form of NO (iNOS) is a common marker of angiogenesis, which resulted in decrease in cells from both lung cancer mice models treated with pomegranate fruit extract. Likewise, a decrease in the number of CD31+ cells was observed, the platelet-derived endothelial cell adhesion protein that represents an index of inhibition of tumor angiogenesis. VEGF expression was also downregulated in mice fed with pomegranate fruit extract and the microvessel density reduced (77.8% in pomegranate treated mice compared to benzo[a]pyrene only-treated mice; 65% in pomegranate treated mice compared to N-nitroso-tris-chloroethylurea only-treated mice) [113].

#### 3.2.4. Colon Cancer

Many studies indicate that pomegranate and its pholyphenols exert a remarkable effect not only in colon cancer chemoprevention but also in chemotherapy, in particular modulating cancer cell death and proliferation.

Pomegranate juice and its constituents such as total pomegranate tannin extract, ellagic acid, and punicalagin were investigated for their* in vitro* antiproliferative and proapoptotic ability on different colon cancer cell lines (SW460, SW620, HT29, and HCT116). Pomegranate juice concentration was normalized to deliver equivalent amounts of punicalagin (w/w). The greatest antiproliferative activity was observed for the juice, for which a 30% to 100% inhibition of cell proliferation was observed at concentrations between 12.5 and 100 *μ*g/mL [3]. Of note, ellagic acid, punicalagin, and total pomegranate tannin extract induced apoptosis in HT29 and HCT116 colon cancer cells at the concentration of 100 *μ*g/mL but failed if cells were treated at doses held equivalent to that detected in pomegranate juice [3]. Again, this underlies the fact that the biological effects of pomegranate come from the synergistic action of pomegranate polyphenols, including flavonols and anthocyanins. Furthermore, on HT29 colon cancer cells, pomegranate juice, ellagic acid, total pomegranate tannin extract and punicalagin induced apoptosis, whereas in the HCT116 colon cancer cells ellagic acid, punicalagin and total pomegranate tannin extract (all at 100 *μ*g/mL) induced apoptosis, but pomegranate juice did not [3]. This evidence might be attributed to the different extent of differentiation of these two cell lines: HCT116 is a highly aggressive cell line unable to differentiate and HT29 retains the ability to differentiate [114].

Pomegranate juice derived ellagitannins (gallic acid, a dimer of gallic acid, ellagic acid, punicalins, and punicalagins) and the intestinal metabolites urolithins exhibited dose- and time-dependent decrease of cell proliferation on HT29 mediated by cell-cycle arrest and followed by the induction of cell death. Among the analyzed ellagitannins, gallic acid dimer was the most effective in the inhibition of proliferation (IC_50_ = 123 *μ*M), also after short time of treatment (12–24 h), whereas the other compounds exhibited antiproliferative effects between 24 and 48 h. Ellagic acid became less effective with an IC_50_ of 462 *μ*M [115]. Cell-cycle arrest induced by ellagitannins was mainly represented by a block in S phase, confirmed by the downregulation of cdk A and B1, necessary for cell progression to G2/M phase. Also urolithins induced a cell-cycle block, but in G2/M phase [115], probably related to its modulation of MAPK, as observed in previous experiments in Caco2 cells [115]. Furthermore, the block of cell cycle in the S phase by ellagic acid and punicalagin, with activation of cyclin E and downregulation of cyclins A and B1, is in accordance with the effects observed in Caco2 cells [13].

The nature of pomegranate-induced cell death in human colon cancer cells was defined. Both ellagitannins and urolithins induced apoptosis by the dose-dependent activation of caspase-3, with the highest induction recorded for punicalagin 100 *μ*M (197% compared to control) [115]. The proapoptotic activity of punicalagin and ellagic acid was Fas- and caspase-8-independent, while the activation of caspase-3 and caspase-9, the release of cytochrome c in the cytosol, and the downregulation of Bcl-XL confirmed the involvement of the apoptotic intrinsic pathway. The effects were recorded at the highest tested concentrations (30 *μ*M for ellagic acid and 100 *μ*M for punicalagin) [13]. Of note, the proapoptotic effect of punicalagin and ellagic acid observed in colon cancer cells was not recorded in normal colon cells [13], thus suggesting a selective action towards cancer cells.

#### 3.2.5. Leukemia

The potential effect of pomegranate juice extract on the inhibition of cell proliferation and induction of apoptosis has been investigated also in leukemia cells. On different leukemia cell lines (four lymphoblastic (Jurkat, SUP-B15, MOLT-3, and CCRF-CEM) and four myeloblastic (HL-60, THP-1, K562, and KG1a) cell lines), pomegranate juice extract (6.25% and 12.5% (v/v)) significantly induced apoptosis. In particular, lymphoblastic cells became the most sensitive to pomegranate, with 2.1% viable cells recorded in CCRF-CEM after treatment with pomegranate 6.25% for 24 h and 0.02% after treatment with pomegranate 12.5%. KG-1a was the most sensitive among the myeloblastic cell lines (31% viable cells after 6.25% pomegranate juice extract treatment and 6.25% viable cells after 12.5%), whereas THP-1 was the less affected by pomegranate (63% viable cells at the maximum tested concentration). The viability of nontumor hematopoietic stem cells (HSCs) was also affected by pomegranate juice extract but at a less extent compared to the majority of the leukemic cells analyzed [116]. The different sensitivity to pomegranate juice extract of lymphoblastic and myeloblastic cells is not surprising and may be justified by the different molecular pathways altered in those two types of leukemia. These differences are often exploited to obtain antileukemic drugs specific for lymphoblastic rather than myeloblastic leukemia [117, 118]. Other natural compounds showed similar effects. For instance, sulforaphane, a dietary isothiocyanate found in cruciferous, induced apoptosis in different types of leukemia blasts but at a different extent [119].

Pomegranate juice extract exhibited antiproliferative effects on all tested cell lines, including nontumor cells. Treatment with pomegranate at the highest tested dose (12.5% (v/v)) caused a significant S phase arrest in all leukemia cell lines, with the exception of HL-60 and KG-1a, where a small % of cells were blocked in the G0/G1 phase [116]. The different modulation of cell-cycle arrest in HL-60 and KG-1 cells confirms a different effect of pomegranate juice extract in myeloblastic leukemia cells as compared to lymphoblastic leukemia cells, possibly imputable in this case to a different modulation of c-myc expression, overexpressed in HL-60 and whose inhibition has been found to be frequently involved in the G0/G1 block [120]. At the lowest pomegranate juice extract tested concentration (6.25% (v/v)), all cells showed a cell-cycle arrest in G0/G1 phase and the induction of senescence, despite being nonsignificant for all cell lines. Nontumor HSCs showed a behavior similar to leukemic cells [116].

Some different fractions of pomegranate extract have been also tested on leukemia cell lines. Five different fractions of pomegranate juice obtained by solid phase extraction were tested on different leukemia cells (CCRF-CEM, MOLT-3, HL-60, and THP-1) to elucidate which constituents are responsible for the pomegranate antileukemic activity [121]. The acetonitrile fraction has been identified as the responsible for apoptosis induction, cell-cycle arrest, and inhibition of cell proliferation. Acetonitrile fraction was the only one able to decrease ATP levels in leukemia cells. The induction of apoptosis by the acetonitrile fraction, tested at 6.25, 12.5, and 25% (v/v), was in line with the results previously obtained with pomegranate juice extract in terms of cell line sensitivity, with CCRF-CEM becoming the most sensitive and THP-1a the least sensitive. Dose-dependent induction of caspase-3 and nuclear morphology characteristics confirmed the above reported evidence. Acetonitrile fraction also induced a block in S phase with a concomitant decrease of cells in G0/G1 phase in all four cell lines after 48 h of treatment. The acetonitrile fraction became the richest in polyphenols and the HPLC analysis indicated the presence of ellagitannins and ellagic acid and the lack of anthocyanins. Punicalagin was the most active among ellagitannins [121]. Moreover, ellagic acid (25 *μ*M) was found to induce caspase-3-dependent apoptosis and S phase cell-cycle block on the promyelocytic cell line (HL60) [122]. Taken together, these findings confirm that the phenolic components are responsible for the main effects induced by pomegranate in leukemia cells including apoptosis, cell-cycle arrest, and inhibition of cell proliferation.

Another mechanism by which pomegranate constituents impart an antileukemic effect has been reported by Kawaii and Lansky [123]. One of the main characteristics of leukemia is the block of cell differentiation at an early stage. The induction of differentiation is an antileukemic strategy with favorable outcomes for substances such as all-trans retinoic acid. Because of the similar structure between plant flavonoids and differentiation-promoting drugs such as retinoids, it was hypothesized that flavonoid-rich pomegranate extracts might have a similar effect on differentiation. Thus, flavonoid-rich fresh and fermented pomegranate juice and aqueous extracts of pomegranate pericarps were evaluated as potential cytodifferentiating agents. Fermented pomegranate juice and pericarp strongly promoted the differentiation of human HL-60 promyelocytic leukemia cells, while fresh juice showed a milder effect [123]. The milder effect of fresh juice could be attributed to the fact that flavonoids are presumably bound to sugar moieties, whereas after fermentation they are free.

Interestingly, ellagic acid (25 *μ*M) enhanced retinoic acid-induced differentiation of promyelocytic leukemic cells towards granulocytic phenotype [122]. Thus, the association of retinoic acid with ellagic acid might be a promising strategy to reduce the therapeutic dosage of retinoic acid and its cardiorespiratory toxicity [124].

Taking into account the promising antileukemic activity of pomegranate, nanoparticles constituted by partially purified pomegranate ellagitannins and gelatin were produced to potentially increase bioavailability and bioactivities. Comparing the proapoptotic ability of pomegranate purified ellagitannins with that of pomegranate purified ellagitannin gelatin nanoparticle suspension in promyelocytic leukemia cells, ellagitannins encapsuled in nanoparticles were less effective than pomegranate purified ellagitannins in the induction of the early stage of apoptosis, while having similar effects in the induction of late stage of apoptosis [125]. Once embedded in nanoparticles, the effect of an active component might be altered [126], but the differences in activity are principally imputable to nanoparticles' cell uptake that depends on particle size, zeta-potential, and morphology. Even if the functionalized punicalagin-nanoparticles seem to respect the criteria for ideal absorption, the apoptotic activity was lower [125]. Further studies are necessary to optimize the formulation of ellagitannin nanoparticles.

#### 3.2.6. Bladder Cancer

Urinary bladder urothelial carcinoma represents the most frequent cancer affecting the urinary system [75]. Taiwanese pomegranate ethanol extract (50 and 100 *μ*g/mL) inhibited the proliferation of T24 and J82 bladder carcinoma cell lines. The inhibition of cell proliferation induced by pomegranate ethanol extract was related to the induction of S phase block, supported by the inhibition of cyclin A protein level and cdk1 expression. The authors investigated the mechanisms underlying the proapoptotic effect induced by the extract in T24 cells. Pomegranate ethanol extract induced the activation of procaspase-3, procaspase-8, and procaspase-9 and the increase in Bax/Bcl-2 ratio, thus proving that apoptosis in T24 cells was induced by pomegranate through the modulation of both intrinsic and extrinsic pathways. Moreover, pomegranate ethanol extract stimulated procaspase-12 and increased the expression of endoplasmic reticulum stress markers, including CHOP and Bip. This evidence suggests that the endoplasmic reticulum stress may play a crucial role in the proapoptotic effect of pomegranate ethanol extract [127].

#### 3.2.7. Brain Tumors

Gliomas are the most frequent brain tumors, with still poor prognosis because of their resistance to surgical and medical treatments. Interestingly, punicalagin (1–30 *μ*g/mL) has been found to induce cell death in U87MG human glioma cells [128]. The decrease in cell viability was associated with an increased expression of cyclin E and decreased expression of cyclins A and B. Punicalagin induced apoptosis in U87MG, as shown by the increase in caspase-9 and caspase-3 activity and PARP cleavage. Apoptosis is not the only mechanisms of cell death induced by punicalagin. Indeed, pretreatment with the caspase inhibitor z-DEVD-fmk did not completely prevent cell death. Accordingly, punicalagin caused autophagic cell death, as confirmed by the increased LC3-II cleavage. Although the role of AMPK in determining autophagy remains to be verified, ectopic expression of p27(Kip1) or phosphorylation on Thr 198 increase autophagy. Accordingly, punicalagin raised the level of phosphorylated AMPK and phosphorylated p27 at Thr198. The dose-dependent decrease in punicalagin-induced cell death after chloroquine treatment, a suppressor of autophagy, further confirmed the ability of punicalagin to induce autophagic cell death [128]. These data, albeit preliminary, are encouraging and justify further investigations on the antitumor activity of punicalagin in gliomas.

## 4. Human Clinical Studies

Copious evidence from preclinical studies indicates the efficacy of pomegranate and its polyphenols against cancer and supports their further development for clinical applications. Interestingly, different clinical studies for human testing of pomegranate have been conducted.

A randomized, placebo-controlled study explored the ability of orally administered 2 g pomegranate extract (delivering pomegranate polyphenols in an amount equivalent to about 250 mL of pomegranate juice) daily for up to 4 weeks to reduce the level of an oxidative stress biomarker (i.e., 8-hydroxy-2′-deoxyguanosine) in 70 patients with a histologic diagnosis of prostate adenocarcinoma [110]. The study evidenced the accumulation of pomegranate extracts in benign and malignant prostate tissues. Furthermore, in benign and cancer tissues, 8-hydroxy-2′-deoxyguanosine levels were 16% and 23% lower in the pomegranate-treated arm, respectively. In both cases, the differences did not reach statistical significance. Taking into account the presumed importance of oxidative stress in prostate cancer development and progression, further and larger studies with longer duration and higher doses are needed to formally test whether pomegranates can alter oxidative stress biomarker levels and its clinical relevance. This is particularly true in that the study found a strong trend between higher urolithin A levels in prostate tissues and lower 8-hydroxy-2′-deoxyguanosine levels.

Several phase II clinical trials have linked oral consumption of pomegranate juice with significant prolongation of PSA doubling time for men with prostate carcinoma with no accompanying serious adverse effects.

In a phase II clinical trial, Pantuck et al. conducted a single-arm phase II trial in 46 patients with recurrent prostate cancer and rising PSA, no prior hormonal therapy, and no evidence of metastases. Patients received 250 mL of pure pomegranate juice (POM Wonderful, containing 570 mg total polyphenol gallic acid equivalents) daily until disease progression. Data on the quantity of punicalagin and ellagic acid were not stated. Although a proper placebo control was not included in the trial, a statistically significant prolongation of PSA doubling time from 15 months at baseline to 54 months after treatment was observed. In addition, a 40% reduction in serum oxidative state was observed in patients. No serious adverse effects were reported [129]. An* in vitro* arm of the trial using patient serum, collected at 9 months after study initiation and incubated with a human prostate cancer cell line, demonstrated that pomegranate juice induced a 12% decrease in cell growth and 17% increase in apoptosis [129].

The above reported finding was further supported by a nonblinded phase II trial in patients with rising PSA and without metastases randomized to receive 1 or 3 g daily of pomegranate extract (containing about 370 mg punicalagin and 30 mg ellagic acid* per* day by HPLC measurement [77]) for up to 18 months [130]. Although a proper placebo control was not included in the trial, the study found that 76% to 82% of patients receiving pomegranate in both arms had longer PSA doubling times values than pretreatment PSA doubling times (from 11.9 to 18.8 months in the low-dose group and 12.2 to 17.5 months in the high-dose group), with no differences in PSA doubling times between arms.

Stenner-Liewen et al. recently investigated the therapeutic impact of pomegranate juice as an adjunct intervention compared to placebo in 98 patients with more advanced or metastatic prostate cancer, the majority (68%) of which had castration-resistant prostate cancer. During the study, patients had to continue their baseline treatment (e.g., androgen deprivation, and zoledronic acid). They consumed 500 mL of pomegranate juice (containing 1147 mg/day polyphenol gallic acid) or 500 mL of placebo beverage daily for 4 weeks. Thereafter, all patients received 250 mL of the pomegranate juice daily (containing 573 mg/day polyphenol gallic acid) for another 4 weeks. Consumption of pomegranate juice did not result in significant PSA declines compared to placebo [131]. There are several differences between the study by Stenner-Liewen et al. [131] and the previous studies reported [129, 130]: (1) the different prostate cancer stage: early prostate cancer stage have been enrolled in the Paller's study and Pantuck's study, while patients with advanced prostate cancer have been enrolled in the Stenner-Liewen's study; (2) PSA levels of patients recruited in the Pantuck's study were 0.5–5 ng/mL versus >5 ng/mL in the study by Stenner-Liewen et al.; (3) the duration of treatment was 12 months in the Pantuck's study, 18 months in the Paller's study, and 2 months in the Stenner-Liewen's study; (4) the observation period was 18 months/progression in the Pantuck's study and Paller' study and 2 months in the Stenner-Liewen's study; (5) taking as a benchmark the daily dosage of 400 mg (370 mg punicalagin and 30 mg ellagic acid) of the Paller's study, the daily pomegranate polyphenol content used by Stenner-Liewen et al. was 20 times (study period) to 40 times (follow-up period) below the required amount for successful anticancer treatment and far too low to produce a clinical effect [132]. Thus, a careful characterization of the active principles should be mandatory prior to performing more well-controlled human studies aimed at demonstrating the efficacy of pomegranate and its polyphenols and provide a deeper understanding of their therapeutic potential in metastatic prostate cancer treatment.

On the whole, the hitherto published clinical trials on prostate cancer seem to suggest that pomegranate might be useful in early and well differentiated prostate cancer and provide important information for the design and patient selection for further trials with pomegranate.

## 5. Final Considerations

A number of studies, both* in vitro* and* in vivo*, demonstrated the ability of pomegranate and its polyphenols to contrast various biological events involved in cancer pathogenesis and progression. This scenario paves the way to a double exploitation of pomegranate polyphenols in cancer: as a chemopreventive strategy to reduce the onset of tumors through diet and direct anticancer agents to treat different human cancers at higher dosage regimens more reliably achievable through pharmaceutical delivery of purified compounds.

Pomegranate contains a mixture of phenols, flavonoids, anthocyanins, and tannins able to modulate cellular biochemistry. It is, therefore, difficult to carefully assess the underlying mechanisms that are responsible for its effects and associate them with a single constituent. Each of these constituents could be targeting a different pathway. Most probably, different components of pomegranate modulate different pathways, thus inhibiting several pathways at the same time. This can be highly effective in treating complex diseases such as cancer, characterized by the deregulation of multiple aberrant signalling pathways.

To better understand the mechanism of pomegranate, it is helpful to assess what levels can be reliably attainable in patients. The bioavailability of flavonoids has yet to be unanimously agreed upon. Some studies report that they are poorly absorbed in the upper gastrointestinal tract. Small intestinal absorption can range from 0% to 60% of the administered dose, dependent upon the food source [133]. It has been found that 240 mL* per* day of pomegranate juice (POM Wonderful, standardized to 570 mg of total polyphenol gallic acid equivalents* per* day) used in the Pantuck's study achieved serum ellagic acid concentrations of 0.14 ± 0.05 *μ*mol/L [129, 134]. However, another study reported concentrations of ellagic acid of only 0.06 *μ*mol/L 1 hour after consuming 180 mL of pomegranate juice [135]. Those differences could be attributed to polymorphisms in metabolic enzymes such as uridine 5′-diphospho-glucuronosyltransferases. Another possibility is whether or not ellagic acid represents the optimal metabolite to measure [136]. Thus, whether the high concentrations used for some pomegranate's polyphenols in the* in vitro* study reflect that which would be found* in vivo* is yet to be elucidated.

Because chemopreventive interventions are aimed at healthy populations at high risk for cancer development, it is important to define the toxicological profile of pomegranate. In rats and mice, oral LD_50_ (median lethal dose) for pomegranate peel extract was greater than 5 g/kg b.w. and the intraperitoneal LD_50_ in rats and mice was 217 and 187 mg/kg b.w., respectively. NOAEL (no observed effect level) for pomegranate peel extract following 90-day administration to rats was 600 mg/kg/day [137]. No changes were observed for blood parameters and serum enzymes in 86 overweight human volunteers after oral ingestion of 1420 mg/day (870 mg gallic acid equivalents) of pomegranate fruit extract for a 28-day period [138]. Another study in 10 patients with carotid artery stenosis did not record any toxic effect for kidney, liver, and heart function after pomegranate juice consumption (121 mg/L ellagic acid equivalents) for up to 3 years [139].

Some studies investigated the toxic profile of pomegranate polyphenols. High doses of punicalagin or punicalin (12.5 and 25 mg/kg b.w.) exerted detrimental effects in rat characterized by serum alanine aminotransferase and aspartate aminotransferase increase, hepatic injury, and increase in hepatic lipid peroxidation [140]. However, a recent study [141] did not report tissue alterations or changes in serum biochemical and hematological parameters in rats upon consumption of a 6% punicalagin-containing diet (approximately equivalent to 350 g/day of punicalagin for a 70 kg person [142]) for 37 days.

An* in vitro* and* in vivo* study dealing with the possible genotoxic effect of a pomegranate whole fruit extract was recently performed [143]. The extract induced point mutations (statistically significant at concentrations ≥2 mg/plate in the Ames test and ≥1.5 mg/mL in the* Saccharomyces cerevisiae* assay), sister chromatid exchanges (statistically significant at concentrations ≥110 *μ*g/mL) and chromosome aberrations (statistically significant at concentrations ≥45 *μ*g/mL)* in vitro*. A significant genotoxic effect (statistically significant at doses ≥70 mg/kg b.w.) was recorded also* in vivo* in both sexes of mouse. Furthermore, a very recent study reported an elevated frequency of *γ*-H2AX foci (i.e., a sensitive measure of DNA double-strand breaks) in mammary cancer cells after treatment with 50 *μ*g/mL of pomegranate extract [61]. Four potentially mutagenic pyridine alkaloids (i.e., pelletierine, pseudopelletierine, iso-pelletierine, and methylisopelletierine) could account for the genotoxic activity of pomegranate [143, 144].

Genotoxicity is implicated in cancer initiation and in the pathogenesis of different chronic degenerative diseases such as atherosclerosis [145], glaucoma [146], and neurodegenerative diseases [147]. Moreover, the dose-response relationship for genotoxic compounds suggests the lack of a threshold. This means that human exposure to genotoxic agents poses a risk at any level. The genotoxic effects reported for pomegranate raised certain concerns over its safety. Further investigations should be undertaken to evaluate the extent to which pomegranate or its components can be consumed without risk to human health and accurately assess the risk/benefit. Thus, caution should be exercised when suggesting the use of pomegranate or its polyphenols for cancer-related therapeutic purposes.
